# The Endocannabinoid System and Oligodendrocytes in Health and Disease

**DOI:** 10.3389/fnins.2018.00733

**Published:** 2018-10-26

**Authors:** Alexander A. Ilyasov, Carolanne E. Milligan, Emily P. Pharr, Allyn C. Howlett

**Affiliations:** ^1^Graduate Program in Neuroscience, Wake Forest School of Medicine, Winston Salem, NC, United States; ^2^Department of Physiology and Pharmacology and Center for Research on Substance Use and Addiction, Wake Forest School of Medicine, Winston-Salem, NC, United States; ^3^Department of Neurobiology and Anatomy, Wake Forest School of Medicine, Winston-Salem, NC, United States; ^4^Department of Neurology and Comprehensive Multiple Sclerosis Center, Wake Forest School of Medicine, Winston-Salem, NC, United States

**Keywords:** 2-arachidonoylglycerol (2-AG), CP55940, HU210, multiple sclerosis (MS), neural stem cells (NSCs), oligodendrocyte precursor cells (OPCs), SR141716, WIN55212-2

## Abstract

Cannabinoid-based interventions are being explored for central nervous system (CNS) pathologies such as neurodegeneration, demyelination, epilepsy, stroke, and trauma. As these disease states involve dysregulation of myelin integrity and/or remyelination, it is important to consider effects of the endocannabinoid system on oligodendrocytes and their precursors. In this review, we examine research reports on the effects of the endocannabinoid system (ECS) components on oligodendrocytes and their precursors, with a focus on therapeutic implications. Cannabinoid ligands and modulators of the endocannabinoid system promote cell signaling in oligodendrocyte precursor survival, proliferation, migration and differentiation, and mature oligodendrocyte survival and myelination. Agonist stimulation of oligodendrocyte precursor cells (OPCs) at both CB_1_ and CB_2_ receptors counter apoptotic processes via Akt/PI3K, and promote proliferation via Akt/mTOR and ERK pathways. CB_1_ receptors in radial glia promote proliferation and conversion to progenitors fated to become oligodendroglia, whereas CB_2_ receptors promote OPC migration in neonatal development. OPCs produce 2-arachidonoylglycerol (2-AG), stimulating cannabinoid receptor-mediated ERK pathways responsible for differentiation to arborized, myelin basic protein (MBP)-producing oligodendrocytes. In cell culture models of excitotoxicity, increased reactive oxygen species, and depolarization-dependent calcium influx, CB_1_ agonists improved viability of oligodendrocytes. In transient and permanent middle cerebral artery occlusion models of anoxic stroke, WIN55212-2 increased OPC proliferation and maturation to oligodendroglia, thereby reducing cerebral tissue damage. In several models of rodent encephalomyelitis, chronic treatment with cannabinoid agonists ameliorated the damage by promoting OPC survival and oligodendrocyte function. Pharmacotherapeutic strategies based upon ECS and oligodendrocyte production and survival should be considered.

## Introduction

Phytocannabinoid use in management of multiple sclerosis (MS) symptoms (Consroe et al., 1997) has led to clinical trial evidence for the efficacy of tetrahydrocannabinol (THC)/cannabidiol (CBD) oromucosal spray (Sativex) in controlling spasticity and pain (Wade et al., 2010; Giacoppo et al., 2017). MS, a demyelinating disease characterized by persistent neuroinflammation and progressive central nervous system (CNS) demyelination (Kutzelnigg and Lassmann, 2014), is only one of many demyelinating neurodegenerative diseases involving oligodendrocytes, the myelinating cells of the CNS. The endocannabinoid system (ECS) (Howlett et al., 2002; Pertwee et al., 2010) involvement in neuroprotection (Panikashvili et al., 2001; van der Stelt and Di Marzo, 2005; Martinez-Orgado et al., 2007; Sanchez and García-Merino, 2012) and the immune system in CNS diseases (Croxford and Yamamura, 2005; Rom and Persidsky, 2013; Chiurchiu et al., 2015; Olah et al., 2017) have been reviewed. Here, we address ECS effects on oligodendrocytes and their precursors, in order to evaluate the evolving research around cannabinoids in healthy development and in demyelinating neurodegenerative diseases.

## Cannabinoids, Oligodendrocyte Precursor Cells and Oligodendrocytes in Health

Oligodendrocytes, myelinating cells of the vertebrate CNS, enable neurons to signal more energy-efficiently and at higher speed due to saltatory conduction, and maintain axonal integrity through trophic and metabolic support (Michalski and Kothary, 2015; Simons and Nave, 2015). Generation of oligodendrocytes is an ongoing process, starting in embryonic development and continuing throughout life (Baumann and Pham-Dinh, 2001; Trotter et al., 2010; Dimou and Gallo, 2015; Michalski and Kothary, 2015). In brief, oligodendrocyte precursor cells (OPCs), also known as NG2-glia, O-2A progenitors, polydendrocytes, or synantocytes, arise from neural stem cells (NSCs), and preferentially populate distinct areas of the developing CNS in lineage- and time-specific waves (Richardson et al., 2006). Upon arrival, many undergo apoptosis, while many others either mature into myelinating oligodendrocytes or persist as progenitors and remain capable of self-renewal as well as production of mature oligodendrocytes well into adulthood (Dawson et al., 2003). These progenitors become distributed throughout gray and white matter and maintain their respective domains by continuously sampling their environment, able to expand to neighboring areas vacated by other OPCs (Kirby et al., 2006; Hughes et al., 2013).

**FIGURE 1:**
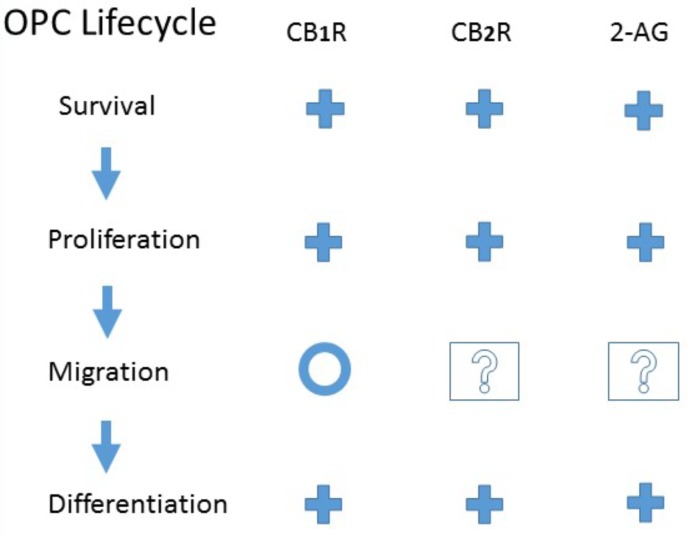
Evidence for CB_1_ or CB_2_ receptor activation or endogenous 2-Arachidonoylglycerol modulation of Oligodendrocyte Precursor Cell Life Cycle. **+**, Promote; **O**, No effect; **?**, Unknown. See the text for details and original references.

Despite this interchangeability, it is becoming increasingly clear that oligodendrocyte precursors represent a heterogeneous group, distinct in their origin, signaling, and ability to revert differentiation to produce neurons and astrocytes (Trotter et al., 2010; Dimou and Gallo, 2015; Nishiyama et al., 2016; Vigano and Dimou, 2016). Regardless of the differences, all OPCs rely on the processes of proliferation, migration, and differentiation to become mature, functioning oligodendrocytes (Baumann and Pham-Dinh, 2001; Miron et al., 2011; Dubois et al., 2014; Sampaio-Baptista and Johansen-Berg, 2017). As these steps are differentially regulated (Marinelli et al., 2016), it is important to look at the effects of cannabinoid agonists on each (see Figure 1).

### Survival

Reports of cannabinoid receptors in newborn rat white matter by immunostaining (Berrendero et al., 1999), led to subsequent studies exploring cells *in vitro*. OPCs were isolated from newborn Wistar rat forebrains and expanded by incubation in serum-free defined media with supplements including platelet-derived growth factor (PDGF) and fibroblast growth factor (FGF) (Molina-Holgado et al., 2002). OPCs could be differentiated into myelin basic protein (MBP)-producing mature oligodendrocytes by incubation in serum-free defined media in which PDGF and FGF were replaced by triiodothyronine (T3). As ascertained by RT-PCR, Western blot, and immunohistochemistry, both OPC and mature oligodendrocytes expressed both CB_1_ and CB_2_ cannabinoid receptors (Molina-Holgado et al., 2002). Because activation of cannabinoid receptors confers neuroprotection (van der Stelt and Di Marzo, 2005; Martinez-Orgado et al., 2007; Sanchez and García-Merino, 2012), the influence of cannabinoid agonists on viability of OPCs was investigated. Upon incubation in serum-free DMEM/F12 media for 12 h, nearly half of OPCs underwent apoptosis (Molina-Holgado et al., 2002). However, most were rescued by concurrent supplementation with CB_1_ agonist arachidonyl-2′-chloroethylamide (ACEA, 25 nM) or CB_1_/CB_2_ agonists WIN55212-2 (25 nM) or HU210 (500 nM). Co-treatment with CB_1_ antagonist SR141716 (1 μM) abolished the anti-apoptotic effect of ACEA, but not of WIN55212-2 or HU210. Both SR141716 plus CB_2_ antagonist SR144528 (1 μM) were required to nullify the pro-survival effect of HU210. These results show that the activation of either CB_1_ or CB_2_ receptors could be sufficient in promoting OPC survival under conditions of trophic factor deprivation. The mechanism includes activation of the phosphatidylinositol 3-kinase (PI3K)/Akt pathway, a known modulator of OPC survival (Vemuri and McMorris, 1996; Ebner et al., 2000). Applying each of the three agonists correlated with increased Akt phosphorylation, while co-treatment with PI3K inhibitors LY294002 (10 μM) or wortmannin (100 nM) nullified the effects of WIN55212-2 and HU210 on both Akt phosphorylation and cell survival (Molina-Holgado et al., 2002). Thus, stimulation of Akt/PI3K pathways via CB_1_ and CB_2_ receptors present in OPCs can curtail apoptotic processes and promote survival.

### Proliferation

Molina-Holgado and colleagues explored the ability of the endocannabinoid system to modulate OPC proliferation and self-renewal (Gomez et al., 2015). In cultured OPCs (Molina-Holgado et al., 2002), 24-h blockade of CB_1_ receptors with AM281 (1 μM), CB_2_ receptors with AM630 (1 μM), or synthesis of 2-arachidonoylglycerol (2-AG) by diacylglycerol lipase α and β (DAGLs) with RHC80267 (5 μM), led to reduction in PDGF/FGF-stimulated OPC proliferation. In the absence of PDGF and FGF, OPC proliferation increased in response to 24-h application of CB_1_ agonist ACEA (0.5 μM), CB_2_ agonist JWH133 (0.5 μM), 2-AG (1 μM), or JZL184 (1 μM) which blocks the 2-AG metabolizing enzyme monoacylglycerol lipase (MAGL). The effect depended on phosphorylation of Akt and mammalian target of rapamycin (mTOR), as their blockers LY294002 (5 μM) and rapamycin (3 nM), respectively (24 h), decreased the proliferative effects of ACEA, JWH133, and JZL184. Extracellular signal regulated-kinase (ERK) 1/2 phosphorylation was decreased in cells treated with the 2-AG synthesis inhibitor RHC80267, as well as with CB_1_ and CB_2_ antagonists AM281 and AM630, respectively. These results extend the impact of cannabinoid receptor activation from promoting OPC survival (Molina-Holgado et al., 2002) to increasing proliferation, while also implicating cannabinoid-mediated Akt/mTOR and ERK pathways, known to play a role throughout OPC development to myelination (Gonsalvez et al., 2016; Figlia et al., 2018).

### Migration

NSCs of postnatal subventricular zone (SVZ) emigrate the neurogenic niche in the form of neuroblasts or OPCs (Marshall et al., 2003). In rat CNS, both myelination and SVZ gliogenesis peak at postnatal day (PD) 15 (Bjelke and Seiger, 1989; Hamano et al., 1996), making this an optimal time-point for exploring cannabinoid effects on OPC proliferation and migration from postnatal SVZ. Immunohistochemical analysis of PD7 and PD15 Wistar rat brain revealed that the CB_1_ receptor was primarily expressed by radial glia (Aré-Martín et al., 2007), which can transform to NSCs (Merkle et al., 2004). However, the CB_2_ receptor co-stained with poly-sialylated neural cell adhesion molecule (PSA-NCAM) (Aré-Martín et al., 2007), a marker of migration in OPCs and neuroblasts (Doetsch et al., 1999; Menn et al., 2006). The CB_1_ agonist ACEA (escalating dose 1.25–2.5 mg/kg, SC daily PD1 to PD14) increased PD15 SVZ staining for 4A4, a marker of radial glia proliferation (Howard et al., 2006), and Olig2 (Aré-Martín et al., 2007), which is expressed in glial precursors fated to become oligodendrocytes (Mitew et al., 2014). Similar treatment with CB_2_ agonist JWH056 (escalating dose 2.5–5.0 mg/kg, SC) increased SVZ staining for PSA-NCAM. Activation of both CB_1_ and CB_2_ receptors by WIN55212-2 (escalating dose 2.5–5.0 mg/kg, SC) increased MBP staining in the external capsule, which was inhibited below control values by concurrent administration of either SR141716 (CB_1_) or SR144528 (CB_2_) antagonists (escalating dose 2–4 mg/kg, SC) (Aré-Martín et al., 2007). These results support the involvement of CB_1_ receptors in radial glia proliferation and conversion to oligodendrocytes, but CB_2_ receptors in OPC migration, leading to functional oligodendrocytes in neonatal myelination.

Studies have examined cannabinoid agonist impact on rodent SVZ proliferation beyond the newborn stage. In juvenile (PD35-PD48) Lewis rats, WIN55212-2 (2 mg/kg, IP bid, 2-weeks) increased SVZ BrdU staining, without changing the ratio of progenitors committed to neuronal or OPC fates, or the number of cells undergoing caspase-3 mediated apoptosis (Bortolato et al., 2014). These results are consistent with findings that genetic ablation of CB_1_ receptors decreased progenitor proliferation in adult mouse SVZ (Jin et al., 2004; Kim et al., 2006). In adult mice, cannabidiol (CBD; 3 mg/kg IP daily for 14 days) increased SVZ proliferative markers Ki67 and BrdU staining (Schiavon et al., 2016). However, CBD at 30 mg/kg decreased proliferation markers (Schiavon et al., 2016), highlighting the importance of dose.

### Differentiation to Mature Oligodendrocytes

Molina-Holgado et al. (2002) investigated OPC differentiation using isolated OPCs differentiated with T3 (30 ng/mL, 48 h) in the absence of PDGF/FGF (Gomez et al., 2011). Activating either cannabinoid receptor (ACEA for CB_1_ and JWH133 for CB_2_, 0.5 μM) increased OPC branching and accumulation of MBP (Western blot). The CB_1_/CB_2_ agonist HU210 (0.5 μM) evoked the same responses, which could be abolished by either AM281 (CB_1_) or AM630 (CB_2_; 1 μM). The mechanism for HU210-mediated OPC arborization and production of MBP involved PI3K/Akt and mTOR pathways, as these effects were blocked with LY290042 (2.5 μM) and rapamycin (0.75 nM), respectively. Gomez et al. (2010) found the Western blot level of DAGLs to be higher in OPCs, whereas the level of MAGL was higher in mature oligodendrocytes, culminating in the finding that OPCs accumulated a greater content of 2-AG than mature oligodendrocytes. Levels of anandamide (AEA) were low and did not differ between the cell stages (Gomez et al., 2010). Differentiation, denoted by branching morphology and levels of MBP after 96 h, was increased by MAGL inhibitor JZL184 (1 μM), and decreased by DAGL inhibitor RHC80267 (5 μM), with exogenous 2-AG (2 μM) abolishing the effect of blocking its synthesis. Inhibition of the ERK pathway by the MEK blocker PD98059 (10 μM) abolished Western blot staining for MBP, implicating the ERK pathway in differentiation. In a CB_1_ receptor mRNA-expressing human oligodendrocyte precursor line HOG16, WIN55212-2 (1 μM, 24 h) increased MBP mRNA expression, particularly in cells treated with T3-supplemented differentiating medium (Tomas-Roig et al., 2016). Collectively, these studies suggest that endogenous 2-AG in OPCs triggers the ERK pathway, leading to the maturation of arborized, MBP-producing oligodendrocytes.

## The ECS and OPCs-Oligodendrocytes in Models of Disease

The role of cannabinoids in OPCs and oligodendrocytes under stress has been evaluated in models of insults such as excitotoxicity (Shouman et al., 2006; Bernal-Chico et al., 2015), reactive oxygen species (ROS) toxicity (Ribeiro et al., 2013), KCl-induced depolarization (Mato et al., 2009), as well as in models of stroke (Fernández-López et al., 2010; Sun et al., 2013a,b), spinal cord injury (SCI) (Marsicano et al., 2003; Arevalo-Martin et al., 2010), and demyelination (Solbrig et al., 2010; Ribeiro et al., 2013; Bernal-Chico et al., 2015; Wen et al., 2015; Feliu et al., 2017).

### Cannabinoids and OPC-Oligodendrocytes in Cytopathology Models

The ability of cannabinoid agonists to protect neurons from excitotoxicity is well known (Shen and Thayer, 1998; Hansen et al., 2002; Marsicano et al., 2003; van der Stelt and Di Marzo, 2005). Likewise, ECS involvement in OPC and oligodendrocyte excitotoxicity has recently been explored (Shouman et al., 2006; Bernal-Chico et al., 2015). Newborn (PD5) Sprague Dawley rats received AMPA/kainate agonist bromowillardine (15 μg, IC), immediately followed by AEA (10 mg/kg, IP) (Shouman et al., 2006). AEA significantly increased OPC density within the periventricular white matter lesion after 1 day, and increased MBP staining after 5 days. The ECS in excitotoxicity was also investigated in mature oligodendrocytes (Bernal-Chico et al., 2015). OPCs were isolated from the cerebral cortex of newborn wildtype or CB_1_-KO Sprague-Dawley rats and differentiated with T3 minus growth factors. Oligodendrocytes were exposed to AMPA plus cyclothiazide (10 μM:100 μM) for 15 min, resulting in excessive cytosolic calcium, reactive oxygen species (ROS) production, and cell death (Bernal-Chico et al., 2015). A 30-min pretreatment with CB_1_ agonist ACEA (25 nM), endocannabinoids AEA or 2-AG (1 μM), or MAGL inhibitor JZL184 (25 nM) reduced cell death (Bernal-Chico et al., 2015). No protection occurred in oligodendrocytes lacking CB_1_ receptors, or pretreated with CB_2_ agonist JWH133 (25 nM) or the inhibitor of AEA metabolizing enzyme fatty acid amide hydrolase (FAAH) URB597 (10 nM – 1 μM). These findings implicate endocannabinoids and CB_1_ receptors in improved viability of oligodendrocytes in excitotoxic conditions.

Due to their high metabolic demand, oligodendrocytes are vulnerable to elevated levels of ROS (McTigue and Tripathi, 2008; Roth and Nunez, 2016), although less so than their precursors (Back et al., 1998). To investigate how cannabinoid agonists affect oligodendrocyte survival, precursors isolated from PD2-PD3 Sprague-Dawley rat brains were differentiated by T3 plus ciliary neurotrophic factor for 2 weeks, and exposed to a peroxynitrite generator SIN-1 (1 mM) (Zhang et al., 2006, 2007) for 2 h (Ribeiro et al., 2013). Concurrent treatment with CB_1_/CB_2_ agonists CP55940 (1–3 μM), WIN55212-2 (10 μM), or anandamide/THC hybrid CB52 (3–10 μM), reduced cell death. CB52’s mechanism included activation of CB_2_ receptors, as CB_2_ antagonist AM630 (10 μM), but not CB_1_ antagonist AM281 (10 μM) reduced its effectiveness, while CB_2_ agonists AM1241 and JWH015 partially replicated it (Ribeiro et al., 2013).

The ability of CB_1_ agonists to inhibit depolarization-dependent calcium channels, as observed in neurons (Howlett et al., 2002), was explored in oligodendrocytes (Mato et al., 2009). Precursors were isolated from optic nerve of PD12 Sprague-Dawley rats, differentiated by 2-day incubation in defined media, and exposed to KCl (50 mM, 1 min) to induce depolarization (Mato et al., 2009). Resulting calcium influx was decreased by CB_1_ agonist ACEA (1 μM) or CB_1_/CB_2_ agonists THC (3 μM), CP55940 (3 μM), AEA (3 μM) or 2-AG (3 μM), but not CB_2_ agonist JWH133 (3 μM). Oligodendrocytes from CB_1_-KO mice were less responsive to AEA and ACEA, although not completely unresponsive, suggesting other targets exist such as transient receptor potential vanilloid receptor-1 (TRPV1) (Ross, 2003; Ruparel et al., 2011), expressed in oligodendrocytes (Gonzalez-Reyes et al., 2013). In contrast, CBD (100 nM, 20–30 min) reduced oligodendrocyte viability, in part through an increase in intracellular calcium (Mato et al., 2010). CBD (1 μM, 10 min) increased oligodendrocyte production of ROS, and induced apoptosis through mitochondria-mediated activation of caspase-8 and -9 and their downstream effector caspase-3, as well as poly-(ADP ribose) polymerase-1 (PARP-1), triggered by caspase-independent mitochondrial apoptosis-induced factor (AIF) (Hong et al., 2004).

### ECS and Oligodendrocytes in Stroke and Traumatic Injury

Oligodendrocytes are vulnerable to ischemic conditions and their replacement via OPCs has been found to aid recovery (Dewar et al., 2003; Zhang et al., 2013, 2016). The impact of the ECS was studied in adult (Sun et al., 2013a,b) and neonatal (Fernández-López et al., 2010) rat models of middle cerebral artery occlusion (MCAO). In transient (2 h) MCAO (Sun et al., 2013a), WIN55212-2 (9 mg/kg, IP immediately after reperfusion and daily) increased staining for penumbral OPCs and mature oligodendrocytes at 4, 7, and 14 days post ischemia (DPI), and penumbral myelin density at 14 DPI. WIN55212-2 reduced penumbral expression of caspase-3 in OPCs at 7 DPI, which correlated with reduced ERK1/2 phosphorylation. These effects were ameliorated by CB_1_ antagonist SR141716 (1 mg/kg, IV). In adult male Sprague-Dawley rats, WIN55212-2 (9 mg/kg, IV) was given within 2 h after permanent MCAO, with anoxia damage quantitated after 24 h (Sun et al., 2013b). WIN55212-2 treatment increased OPC proliferation within the penumbra and ipsilateral SVZ, and decreased penumbral OPC expression of tau-1, an oligodendrocyte marker of ischemic stress (Dewar and Dawson, 1995; Irving et al., 1996). Both effects were partially due to CB_1_ receptor activation (Sun et al., 2013b). In a permanent MCAO model in neonatal PD7 Wistar rats (Fernández-López et al., 2010), WIN55212-2 (1 mg/kg, IP daily for 7 days) increased ipsilateral SVZ OPC proliferation and number of penumbral OPCs at 7 DPI. WIN55212-2 increased the number of mature penumbral oligodendrocytes at 14 and 28 DPI, and accelerated complete MBP recovery. Consistent with earlier findings (Goncalves et al., 2008; Bortolato et al., 2014), WIN55212-2 increased SVZ OPC proliferation even in the absence of ischemia. Although preclinical results appear promising for cannabinoid pharmacotherapies for anoxic demyelination, SVZ neurogenic response to stroke varies greatly between rodents and humans (Kahle and Bix, 2013).

The endocannabinoid 2-AG has been shown to be neuroprotective after traumatic brain injury (Panikashvili et al., 2001), and 2-AG is elevated after spinal trauma (Marsicano et al., 2003; Garcia-Ovejero et al., 2009). Thus, the impact of 2-AG on oligodendrocyte survival was explored in a model of contusive SCI generated by a dropped weight in male adult Wistar rats (Arevalo-Martin et al., 2010). 2-AG (5 mg/kg, IP 30 min after injury) preserved myelin integrity and reduced oligodendrocyte death at the epicenter (1 day post-injury), with the same effects seen as far as 10 mm rostral of epicenter (1 and 7 days post-injury). Co-administration of both CB_1_ and CB_2_ antagonists (AM281 and AM630, respectively; 3 mg/kg, IP), but not by either alone, could reverse the effects of 2-AG. These results support the idea that improved oligodendrocyte survival and preserved white matter integrity underlie the cannabinoid-mediated improvement in SCI recovery (Arevalo-Martin et al., 2012).

### ECS and Models of Demyelination

To investigate demyelination in a mouse model of experimental autoimmune encephalomyelitis (EAE), PD49 or PD56 C57BL/6 mice received a single injection of myelin oligodendrocyte glycoprotein peptide (MOG 35-55), followed by injections of pertussis toxin on the day of MOG inoculation and again 2 days afterward (Bernal-Chico et al., 2015). MAGL inhibitor JZL184 (8 mg/kg, IP daily for 3 weeks, starting on day 14 postinoculation) ameliorated the reduction in spinal cord white matter staining (Bernal-Chico et al., 2015). Similar results in the EAE model were achieved by THC (Moreno-Martet et al., 2015), CBD (Giacoppo et al., 2015), cannabigerol quinone (Carrillo-Salinas et al., 2014), and CB_2_ agonist HU308 (Shao et al., 2014). The effects of CB52 on demyelination EAE (Ribeiro et al., 2013) showed that when initiated before symptom development (3 days) or after clinical disease onset (12 or 20 days), CB52 (2 mg/kg, IP daily) ameliorated the loss of staining for spinal cord myelin and mature oligodendrocytes at day-30. In contrast to CB52’s action on cultured oligodendrocytes *in vitro* (Ribeiro et al., 2013), both of its effects *in vivo* were blocked by CB_1_ antagonist AM281 (2 mg/kg), but not by CB_2_ antagonist AM630 (2 mg/kg).

Microglia are an integral part of demyelinating diseases’ neuroimmune complex (Gonzalez et al., 2014). In microglia, CB_1_ receptors are expressed at low levels constitutively; however, CB_2_ receptors become upregulated when microglia become activated (Cabral et al., 2008). Endocannabinoids 2-AG and AEA have been shown to drive microglia toward alternative, anti-inflammatory activation state, M2, and away from classic, pro-inflammatory polarization, M1, which in turn causes microglia to upregulate its own 2-AG synthesizing enzymes (Mecha et al., 2015). Because microglial 2-AG has been shown to promote OPC differentiation (Miron et al., 2013), blocking its degradation could be of use in counteracting demyelination. This has been explored in a mouse model of EAE (Wen et al., 2015), by inhibiting the 2-AG hydrolyzing microglial enzyme ABHD6 (Li et al., 2007; Marrs et al., 2010; Murataeva et al., 2014) with WWL70 (10 mg/kg, IP daily starting at the onset of clinical symptoms on day-11 postinoculation). WWL70 increased cerebral 2-AG at day-21, and ameliorated the loss of staining of spinal cord myelin and mature oligodendrocytes in wildtype mice on day-28 (Wen et al., 2015). These results were not seen in CB_2_-KO mice, nor when WWL70 was co-administered with CB_2_ antagonist AM630 (3 mg/kg), suggesting that microglial 2-AG accumulation is dependent upon CB_2_ receptor signaling. Co-administration with CB_1_ antagonist AM281 failed to interfere with WWL70’s effects.

OPC gliogenesis in Borna Disease Virus (BDV) encephalomyelitis, generated in PD28 male Lewis rats (Solbrig et al., 2010), demonstrated that WIN55212-2 (1 mg/kg, IP daily for 7-days starting 1 week after virus inoculation) increased OPC proliferation in striatum, decreased apoptosis of proliferating cells, skewed precursor differentiation away from astrocytes and toward oligodendrocytes, and promoted OPC maturation. In uninfected controls, WIN55212-2 increased proliferation in both PFC and striatum.

In Theiler’s murine encephalomyelitis virus-induced demyelinating disease (TMEV-IDD), PD28 female CJL/J mice received an intracerebral injection of the Daniel strain virus (Feliu et al., 2017). When started after symptom onset at day-75, a 10-day treatment with MAGL inhibitor UCM03025 (5 mg/kg, IP) increased the spinal cord populations of both mature oligodendrocytes and OPCs, and restored MBP level to that of sham controls (Feliu et al., 2017).

In the cuprizone oligodendrotoxic model (Bernal-Chico et al., 2015), PD56 C57BL/6 mice were fed a cuprizone-supplemented diet (0.3%) for 3 weeks. Concurrent MAGL inhibitor JZL184 (8 mg/kg, IP daily) ameliorated cuprizone-induced reduction in corpus callosum MBP staining (Bernal-Chico et al., 2015), implicating 2-AG-mediated protection.

Seizures are known to accompany demyelination in experimental models (DePaula-Silva et al., 2017; Lapato et al., 2017; Spatola and Dalmau, 2017) as well as MS (Koch et al., 2008; Anderson and Rodriguez, 2011; Sponsler and Kendrick-Adey, 2011). The ECS promotion of OPCs (Solbrig et al., 2010; Feliu et al., 2017) and mature oligodendrocytes (Ribeiro et al., 2013; Wen et al., 2015; Feliu et al., 2017) may counteract demyelination observed in patients with intractable epilepsy (Hu et al., 2016).

### CBD and OPCs in Inflammation

CBD has been promoted for potential therapeutic applications (Devinsky et al., 2014; Blessing et al., 2015; Ibeas Bih et al., 2015) including anti-inflammation (Burstein, 2015). Inflammation underlies a range of pathologies including neurodegeneration (Glass et al., 2010; Cunningham, 2013), stroke (Turner and Vink, 2007; Ahmad et al., 2014), and demyelination (Popescu and Lucchinetti, 2012; Kutzelnigg and Lassmann, 2014). To examine CBD’s anti-inflammatory impact on OPC survival, cultured OPCs isolated from the forebrain of newborn Wistar rats were exposed to inflammation-related stressors (Mecha et al., 2012). Treatment with CBD (1 μM) reduced: (1) caspase-3-mediated apoptosis resulting from lipopolysaccharides (LPS) and interferon-γ (IFNγ)-mediated inflammation (48 h); (2) cell death induced by endoplasmic reticulum stress instigated by tunicamycin (1 μg/ml, 24 h); and (3) cell detachment and ROS production in response to hydrogen peroxide (2 h). CBD was unable to increase OPC proliferation in culture (Mecha et al., 2012), in contrast to its chronic administration in SVZ of adult Swiss mice (Schiavon et al., 2016). CBD did not promote apoptosis in culture, as observed in unstressed cultured oligodendrocytes (Mato et al., 2010). CBD’s cellular mechanism(s) have yet to be established for OPC and oligodendrocyte function, but might counter the endocannabinoid responses at their receptors. Further, CBD may target other cell types in the neuro-immune complex, explaining differences between *in vitro* vs. *in vivo* models.

## Perspectives

Although much has been learned about the impact of cannabinoid agonists on oligodendrocytes in health and disease, many questions remain unexplored, such as the cannabinoid impact on OPC local migration (Kirby et al., 2006; Hughes et al., 2013) and glutamate signaling (Spitzer et al., 2016), the oligodendrocyte’s ability to produce myelin and provide metabolic support to axons (Zecca et al., 2004; Saab et al., 2013; Simons and Nave, 2015). Cannabinoid agonists also comprise structurally and functional distinct ligands (Howlett et al., 2002; Pertwee et al., 2010; Laprairie et al., 2017; Priestley et al., 2017), and as such, it is important to characterize their pharmacological profiles in cell pathologies related to oligodendrocytes and other cell types in the neuro-immune complex. Although the impact of cannabinoid extracts on MS disease progression remains inconclusive (Pertwee, 2007; Pryce et al., 2015), the outlook is optimistic (Arévalo-Martin et al., 2008; Chiurchiu et al., 2015, 2018). Evidence of cannabinoid agonist effects on oligodendrocyte survival and OPC lifecycle suggests their usefulness in CNS pathologies such as demyelination (Popescu and Lucchinetti, 2012; Kutzelnigg and Lassmann, 2014), neurodegeneration (Ettle et al., 2016; Tauheed et al., 2016), ischemia (Dewar et al., 2003; Mifsud et al., 2014), epilepsy (Friedman and Devinsky, 2015; Stockings et al., 2018), and traumatic injuries to spinal cord (Alizadeh and Abdolrezaee-Karimi, 2016; Levine, 2016; Arevalo-Martin et al., 2016) and brain (Armstrong et al., 2016; Takase et al., 2018).

## Author Contributions

AI conceived the idea and approach to the review, wrote and edited the manuscript. CM and EP provided the feedback and edited the manuscript. AH developed approach to the review, structured, wrote and edited the manuscript.

## Conflict of Interest Statement

The authors declare that the research was conducted in the absence of any commercial or financial relationships that could be construed as a potential conflict of interest.

## References

[B1] Ahmad M. Dar N. J. Bhat Z. S. Hussain A. Shah A. Liu H. (2014). Inflammation in ischemic stroke: mechanisms, consequences and possible drug targets. *CNS Neurol. Disord. Drug Targets* 13 1378–1396. 10.2174/1871527313666141023094720 25345517

[B2] Alizadeh A. Abdolrezaee-Karimi S. (2016). Microenvironmental regulation of oligodendrocyte replacement and remyelination in spinal cord injury. *J. Physiol.* 594 3539–3552. 10.1113/JP270895 26857216 PMC4929323

[B3] Anderson G. Rodriguez M. (2011). Multiple sclerosis, seizures, and antiepileptics: role of IL-18, IDO, and melatonin. *Eur. J. Neurol.* 18 680–685. 10.1111/j.1468-1331.2010.03257.x 21118329

[B4] Arévalo-Martín Á. García-Ovejero D. Rubio-Araiz A. Gómez O. Molina-Holgado F. Molina-Holgado E. (2007). Cannabinoids modulate Olig2 and polysialylated neural cell adhesion molecule expression in the subventricular zone of post-natal rats through cannabinoid receptor 1 and cannabinoid receptor 2. *Eur. J. Neurosci.* 26 1548–1559. 10.1111/j.1460-9568.2007.05782.x 17880390

[B5] Arevalo-Martin A. Molina-Holgado E. Garcia-Ovejero D. (2016). Cannabinoids to treat spinal cord injury. *Prog. Neuropsychopharmacol. Biol. Psychiatry* 64 190–199. 10.1016/j.pnpbp.2015.03.008 25805333

[B6] Arévalo-Martin A. Garcia-Ovejero D. Gómez O. Rubio-Araiz A. Navarro-Galve B. Guaza C. (2008). CB_2_ cannabinoid receptors as an emerging target for demyelinating diseases: from neuroimmune interactions to cell replacement strategies. *Br. J. Pharmacol.* 153 216–225. 10.1038/sj.bjp.0707466 17891163 PMC2219542

[B7] Arevalo-Martin A. Garcia-Ovejero D. Molina-Holgado E. (2010). The endocannabinoid 2-arachidonoylglycerol reduces lesion expansion and white matter damage after spinal cord injury. *Neurobiol. Dis.* 38 304–312. 10.1016/j.nbd.2010.02.002 20156559

[B8] Arevalo-Martin A. Garcia-Ovejero D. Sierra-Palomares Y. Paniagua-Torija B. Gonzalez-Gil I. Ortega-Gutierrez S. (2012). Early endogenous activation of CB1 and CB2 receptors after spinal cord injury is a protective response involved in spontaneous recovery. *PLoS One* 7:e49057. 10.1371/journal.pone.0049057 23152849 PMC3496738

[B9] Armstrong R. C. Mierzwa A. J. Marion C. M. Sullivan G. M. (2016). White matter involvement after TBI: clues to axon and myelin repair capacity. *Exp. Neurol.* 275 3328–3333. 10.1016/j.expneurol.2015.02.011 25697845

[B10] Back S. A. Gan X. Li Y. Rosenberg P. A. Volpe J. J. (1998). Maturation-dependent vulnerability of oligodendrocytes to oxidative stress-induced death caused by glutathione depletion. *J. Neurosci.* 18 6241–6253. 10.1523/JNEUROSCI.18-16-06241.1998 9698317 PMC6793198

[B11] Baumann N. Pham-Dinh D. (2001). Biology of oligodendrocyte and myelin in the mammalian central nervous system. *Physiol. Rev.* 81 871–927. 10.1152/physrev.2001.81.2.871 11274346

[B12] Bernal-Chico A. Canedo M. Manterola A. Victoria Sanchez-Gómez M. Pérez-Samartin A. Rodriguez-Puertas R. (2015). Blockade of monoacylglycerol lipase inhibits oligodendrocyte excitotoxicity and prevents demyelination *in vivo*. *Glia* 63 163–176. 10.1002/glia.22742 25130621

[B13] Berrendero F. Sepe N. Ramos J. A. Di Marzo V. Fernández-Ruiz J. J. (1999). Analysis of cannabinoid receptor binding and mRNA expression and endogenous cannabinoid contents in the developing rat brain during late gestation and early postnatal period. *Synapse* 33 181–191. 10.1002/(SICI)1098-2396(19990901)33:3<181::AID-SYN3>3.0.CO;2-R 10420166

[B14] Bjelke B. Seiger A. (1989). Morphological distribution of MBP-like immunoreactivity in the brain during development. *Int. J. Dev. Neurosci.* 7 145–164. 10.1016/0736-5748(89)90065-8 2469297

[B15] Blessing E. M. Steenkamp M. M. Manzanares J. Marmar C. R. (2015). Cannabidiol as a potential treatment for anxiety disorders. *Neurotherapeutics* 12 825–836. 10.1007/s13311-015-0387-1 26341731 PMC4604171

[B16] Bortolato M. Bini V. Frau R. Devoto P. Pardu A. Fan Y. (2014). Juvenile cannabinoid treatment induces frontostriatal gliogenesis in Lewis rats. *Eur. Neuropsychopharmacol.* 24 974–985. 10.1016/j.euroneuro.2013.12.011 24630433

[B17] Burstein S. (2015). Cannabidiol (CBD) and its analogs: a review of their effects on inflammation. *Bioorg. Med. Chem.* 23 1377–1385. 10.1016/j.bmc.2015.01.059 25703248

[B18] Cabral G. A. Raborn E. S. Griffin L. Dennis J. Marciano-Cabral F. (2008). CB_2_ receptors in the brain: role in central immune function. *Br. J. Pharmacol.* 153 240–251. 10.1038/sj.bjp.0707584 18037916 PMC2219530

[B19] Carrillo-Salinas F. J. Navarrete C. Mecha M. Feliú A. Collado J. A. Cantarero I. (2014). A cannabigerol derivative suppresses immune responses and protects mice from experimental autoimmune encephalomyelitis. *PLoS One* 9:e94733. 10.1371/journal.pone.0094733 24727978 PMC3984273

[B20] Chiurchiu V. Leuti A. Maccarrone M. (2015). Cannabinoid signaling and neuroinflammatory diseases: a melting pot for the regulation of brain immune responses. *J. Neuroimmune. Pharmacol.* 10 268–280. 10.1007/s11481-015-9584-2 25601726

[B21] Chiurchiu V. van der Stelt M. Centonze D. Maccarrone M. (2018). The endocannabinoid system and its therapeutic exploitation in multiple sclerosis: clues for other neuroinflammatory diseases. *Prog. Neurobiol.* 160 82–100. 10.1016/j.pneurobio.2017.10.007 29097192

[B22] Consroe P. Musty R. Rein J. Tillery W. Pertwee R. (1997). The perceived effects of smoked cannabis on patients with multiple sclerosis. *Eur. Neurol.* 38 44–48. 10.1159/000112901 9252798

[B23] Croxford J. L. Yamamura T. (2005). Cannabinoids and the immune system: potential for the treatment of inflammatory diseases? *J. Neuroimmunol.* 166 3–18. 10.1016/j.jneuroim.2005.04.023 16023222

[B24] Cunningham C. (2013). Microglia and neurodegeneration: the role of systemic inflammation. *Glia* 61 71–90. 10.1002/glia.22350 22674585

[B25] Dawson M. R. Polito A. Levine J. M. Reynolds R. (2003). NG2-expressing glial progenitor cells: an abundant and widespread population of cycling cells in the adult rat CNS. *Mol. Cell. Neurosci.* 24 476–488. 10.1016/S1044-7431(03)00210-0 14572468

[B26] DePaula-Silva A. B. Hanak T. J. Libbey J. E. Fujinami R. S. (2017). Theiler’s murine encephalomyelitis virus infection of SJL/J and C57BL/6J mice: Models for multiple sclerosis and epilepsy. *J. Neuroimmunol.* 308 30–42. 10.1016/j.jneuroim.2017.02.012 28237622 PMC5474355

[B27] Devinsky O. Cilio M. R. Cross H. Fernandez-Ruiz J. French J. Hill C. (2014). Cannabidiol: pharmacology and potential therapeutic role in epilepsy and other neuropsychiatric disorders. *Epilepsia* 55 791–802. 10.1111/epi.12631 24854329 PMC4707667

[B28] Dewar D. Dawson D. (1995). Tau protein is altered by focal cerebral ischaemia in the rat: an immunohistochemical and immunoblotting study. *Brain Res.* 684 70–78. 10.1016/0006-8993(95)00417-O 7583206

[B29] Dewar D. Underhill S. M. Goldberg M. P. (2003). Oligodendrocytes and ischemic brain injury. *J. Cereb. Blood Flow Metab.* 23 263–274. 10.1097/01.WCB.0000053472.41007.F9 12621301

[B30] Dimou L. Gallo V. (2015). NG2-glia and their functions in the central nervous system. *Glia* 63 1429–1451. 10.1002/glia.22859 26010717 PMC4470768

[B31] Doetsch F. Caillé I. Lim D. A. García-Verdugo J. M. Alvarez-Buylla A. (1999). Subventricular zone astrocytes are neural stem cells in the adult mammalian brain. *Cell* 9 703–716. 10.1016/S0092-8674(00)80783-7 10380923

[B32] Dubois J. Dehaene-Lambertz G. Kulikova S. Poupon C. Hüppi P. S. Hertz-Pannier L. (2014). The early development of brain white matter: a review of imaging studies in fetuses, newborns and infants. *Neuroscience* 276 48–71. 10.1016/j.neuroscience.2013.12.044 24378955

[B33] Ebner S. Dunbar M. McKinnon R. D. (2000). Distinct roles for PI3K in proliferation and survival of oligodendrocyte progenitor cells. *J. Neurosci. Res.* 62 336–345. 10.1002/1097-4547(20001101)62:3<336::AID-JNR3>3.0.CO;2-H 11054802

[B34] Ettle B. Schlachetzki J. C. M. Winkler J. (2016). Oligodendroglia and myelin in neurodegenerative diseases: more than just bystanders? *Mol. Neurobiol.* 53 3046–3062. 10.1007/s12035-015-9205-3 25966971 PMC4902834

[B35] Feliú A. Bonilla Del Río I. Carrillo-Salinas F. J. Hernandez-Torres G. Mestre L. Puente N. (2017). 2-Arachidonoylglycerol reduces proteoglycans and enhances remyelination in a progressive model of demyelination. *J. Neurosci.* 37 8385–8398. 10.1523/JNEUROSCI.2900-16.2017 28751457 PMC6596867

[B36] Fernández-López D. Pradillo J. M. García-Yébenes I. Martínez-Orgado J. A. Moro M. A. Lizasoain I. (2010). The cannabinoid WIN55212-2 promotes neural repair after neonatal hypoxia-ischemia. *Stroke* 41 2956–2964. 10.1161/STROKEAHA.110.599357 21115947

[B37] Figlia G. Gerber D. Suter U. (2018). Myelination and mTOR. *Glia* 66 693–707. 10.1002/glia.23273 29210103 PMC5836902

[B38] Friedman D. Devinsky O. (2015). Cannabinoids in the treatment of epilepsy. *N. Engl. J. Med.* 373 1048–1058. 10.1056/NEJMra1407304 26352816

[B39] Garcia-Ovejero D. Arevalo-Martin A. Petrosino S. Docagne F. Hagen C. Bisogno T. (2009). The endocannabinoid system is modulated in response to spinal cord injury in rats. *Neurobiol. Dis.* 33 57–71. 10.1016/j.nbd.2008.09.015 18930143

[B40] Giacoppo S. Bramanti P. Mazzon E. (2017). Sativex in the management of multiple sclerosis-related spasticity: an overview of the last decade of clinical evaluation. *Mult. Scler. Relat. Disord.* 17 22–31. 10.1016/j.msard.2017.06.015 29055461

[B41] Giacoppo S. Galuppo M. Pollastro F. Grassi G. Bramanti P. Mazzon E. (2015). A new formulation of cannabidiol in cream shows therapeutic effects in a mouse model of experimental autoimmune encephalomyelitis. *Daru* 23:48. 10.1186/s40199-015-0131-8 26489494 PMC4618347

[B42] Glass C. K. Saijo K. Winner B. Marchetto M. C. Gage F. H. (2010). Mechanisms underlying inflammation in neurodegeneration. *Cell* 140 918–934. 10.1016/j.cell.2010.02.016 20303880 PMC2873093

[B43] Gomez O. Arevalo-Martin A. Garcia-Ovejero D. Ortega-Gutierrez S. Cisneros J. A. Almazan G. (2010). The constitutive production of the endocannabinoid 2-arachidonoylglycerol participates in oligodendrocyte differentiation. *Glia* 58 1913–1927. 10.1002/glia.21061 20878765

[B44] Gomez O. Sanchez-Rodriguez M. A. Ortega-Gutierrez S. Vazquez-Villa H. Guaza C. Molina-Holgado F. (2015). A basal tone of 2-Arachidonoylglycerol contributes to early oligodendrocyte progenitor proliferation by activating phosphatidylinositol 3-kinase (PI3K)/AKT and the mammalian target of rapamycin (MTOR) pathways. *J. Neuroimmune. Pharmacol.* 10 309–317. 10.1007/s11481-015-9609-x 25900077

[B45] Gomez O. Sanchez-Rodriguez A. Le M. Sanchez-Caro C. Molina-Holgado F. Molina-Holgado E. (2011). Cannabinoid receptor agonists modulate oligodendrocyte differentiation by activating PI3K/Akt and the mammalian target of rapamycin (mTOR) pathways. *Br. J. Pharmacol.* 163 1520–1532. 10.1111/j.1476-5381.2011.01414.x 21480865 PMC3165960

[B46] Goncalves M. B. Suetterlin P. Yip P. Molina-Holgado F. Walker D. J. Oudin M. J. (2008). A diacylglycerol lipase-CB2 cannabinoid pathway regulates adult subventricular zone neurogenesis in an age-dependent manner. *Mol. Cell. Neurosci.* 3 526–536. 10.1016/j.mcn.2008.05.001 18562209

[B47] Gonsalvez D. Ferner A. H. Peckham H. Murray S. S. Xiao J. (2016). The roles of extracellular related-kinases 1 and 2 signaling in CNS myelination. *Neuropharmacology* 110 586–593. 10.1016/j.neuropharm.2015.04.024 25959068

[B48] Gonzalez H. Elgueta D. Montoya A. Pacheco R. (2014). Neuroimmune regulation of microglial activity involved in neuroinflammation and neurodegenerative diseases. *J. Neuroimmunol.* 274 1–13. 10.1016/j.jneuroim.2014.07.012 25091432

[B49] Gonzalez-Reyes L. E. Ladas T. P. Chiang C. C. Durand D. M. (2013). TRPV1 antagonist capsazepine suppresses 4-AP-induced epileptiform activity *in vitro* and electrographic seizures *in vivo*. *Exp. Neurol.* 250 321–332. 10.1016/j.expneurol.2013.10.010 24145133 PMC4104534

[B50] Hamano K. Iwasaki N. Takeya T. Takita H. (1996). A quantitative analysis of rat central nervous system myelination using the immunohistochemical method for MBP. *Brain Res. Dev. Brain Res.* 93 18–22. 10.1016/0165-3806(96)00025-9 8804688

[B51] Hansen H. H. Azcoitia I. Pons S. Romero J. Garcia-Segura L. M. Ramos J. A. (2002). Blockade of cannabinoid CB_1_ receptor function protects against *in vivo* disseminating brain damage following NMDA-induced excitotoxicity. *J. Neurochem.* 82 154–158. 10.1046/j.1471-4159.2002.00961.x 12091476

[B52] Hong S. J. Dawson T. M. Dawson V. L. (2004). Nuclear and mitochondrial conversations in cell death: PARP-1 and AIF signaling. *Trends Pharmacol. Sci.* 25 259–264. 10.1016/j.tips.2004.03.005 15120492

[B53] Howard B. Chen Y. Zecevic N. (2006). Cortical progenitor cells in the developing human telencephalon. *Glia* 53 57–66. 10.1002/glia.20259 16158418

[B54] Howlett A. C. Barth F. Bonner T. I. Cabral G. Casellas P. Devane W. A. (2002). International union of pharmacology. XXVII. Classification of cannabinoid receptors. *Pharmacol. Rev.* 5 161–202. 10.1124/pr.54.2.161 12037135

[B55] Hu X. Wang J. Y. Gu R. Qu H. Li M. Chen L. (2016). The relationship between the occurrence of intractable epilepsy with glial cells and myelin sheath - an experimental study. *Eur. Rev. Med. Pharmacol. Sci.* 20 4516–4524. 27874947

[B56] Hughes E. G. Kang S. H. Fukaya M. Bergles D. E. (2013). Oligodendrocyte progenitors balance growth with self-repulsion to achieve homeostasis in the adult brain. *Nat. Neurosci.* 16 668–676. 10.1038/nn.3390 23624515 PMC3807738

[B57] Ibeas Bih C Chen T. Nunn A. V. Bazelot M. Dallas M. Whalley B. J. (2015). Molecular targets of cannabidiol in neurological disorders. *Neurotherapeutics* 12 699–730. 10.1007/s13311-015-0377-3 26264914 PMC4604182

[B58] Irving E. A. Nicoll J. Graham D. I. Dewar D. (1996). Increased tau immunoreactivity in oligodendrocytes following human stroke and head injury. *Neurosci. Lett.* 213 189–192. 10.1016/0304-3940(96)12856-1 8873146

[B59] Jin K. Xie L. Kim S. H. Parmentier-Batteur S. Sun Y. Mao X. O. (2004). Defective adult neurogenesis in CB1 cannabinoid receptor knockout mice. *Mol. Pharmacol.* 66 204–208. 10.1124/mol.66.2.204 15266010

[B60] Kahle M. P. Bix G. J. (2013). Neuronal restoration following ischemic stroke: influences, barriers, and therapeutic potential. *Neurorehabil. Neural Repair* 27 469–478. 10.1177/1545968312474119 23392917

[B61] Kim S. H. Won S. J. Mao X. O. Ledent C. Jin K. Greenberg D. A. (2006). Role for neuronal nitric-oxide synthase in cannabinoid-induced neurogenesis. *J. Pharmacol. Exp. Ther.* 319 150–154. 10.1124/jpet.106.107698 16831955

[B62] Kirby B. B. Takada N. Latimer A. J. Shin J. Carney T. J. Kelsh R. N. (2006). In vivo time-lapse imaging shows dynamic oligodendrocyte progenitor behavior during zebrafish development. *Nat. Neurosci.* 9 1506–1511. 10.1038/nn1803 17099706

[B63] Koch M. Uyttenboogaart M. Polman S. De Keyser J. (2008). Seizures in multiple sclerosis. *Epilepsia* 49 948–953. 10.1111/j.1528-1167.2008.01565.x 18336559

[B64] Kutzelnigg A. Lassmann H. (2014). Pathology of multiple sclerosis and related inflammatory demyelinating diseases. *Handb. Clin. Neurol* 122 15–58. 10.1016/B978-0-444-52001-2.00002-9 24507512

[B65] Lapato A. S. Szu J. I. Hasselmann J. P. C. Khalaj A. J. Binder D. K. Tiwari-Woodruff S. K. (2017). Chronic demyelination-induced seizures. *Neuroscience* 346 409–422. 10.1016/j.neuroscience.2017.01.035 28153692 PMC5394933

[B66] Laprairie R. B. Bagher A. M. Denovan-Wright E. M. (2017). Cannabinoid receptor ligand bias: implications in the central nervous system. *Curr. Opin. Pharmacol* 32 32–43. 10.1016/j.coph.2016.10.005 27835801

[B67] Levine J. (2016). The reactions and role of NG2 glia in spinal cord injury. *Brain Res.* 1638 199–208. 10.1016/j.brainres.2015.07.026 26232070 PMC4732922

[B68] Li W. Blankman J. L. Cravatt B. F. (2007). A functional proteomic strategy to discover inhibitors for uncharacterized hydrolases. *J. Am. Chem. Soc.* 129 9594–9595. 10.1021/ja073650c 17629278

[B69] Marinelli C. Bertalot T. Zusso M. Skaper S. D. Giusti P. (2016). Systematic review of pharmacological properties of the oligodendrocyte lineage. *Front. Cell Neurosci.* 10:27 10.3389/fncel.2016.00027 PMC4751280 26903812

[B70] Marrs W. R. Blankman J. L. Horne E. A. Thomazeau A. Lin Y. H. Coy J. (2010). The serine hydrolase ABHD6 controls the accumulation and efficacy of 2-AG at cannabinoid receptors. *Nat. Neurosci.* 13 951–957. 10.1038/nn.2601 20657592 PMC2970523

[B71] Marshall C. A. Suzuki S. O. Goldman J. E. (2003). Gliogenic and neurogenic progenitors of the subventricular zone: Who are they, where did they come from, and where are they going? *Glia* 43 52–61. 12761867 10.1002/glia.10213

[B72] Marsicano G. Goodenough S. Monory K. Hermann H. Eder M. Cannich A. (2003). CB1 cannabinoid receptors and on-demand defense against excitotoxicity. *Science* 302 84–88. 10.1126/science.1088208 14526074

[B73] Martinez-Orgado J. Fernandez-Lopez D. Lizasoain I. Romero J. (2007). The seek of neuroprotection: introducing cannabinoids. *Recent Pat. CNS Drug Discov.* 2 131–139. 10.2174/157488907780832724 18221224

[B74] Mato S. Alberdi E. Ledent C. Watanabe M. Matute C. (2009). CB1 cannabinoid receptor-dependent and -independent inhibition of depolarization-induced calcium influx in oligodendrocytes. *Glia* 57 295–306. 10.1002/glia.20757 18814177

[B75] Mato S. Victoria Sanchez-Gomez M. Matute C. (2010). Cannabidiol induces intracellular calcium elevation and cytotoxicity in oligodendrocytes. *Glia* 58 1739–1747. 10.1002/glia.21044 20645411

[B76] McTigue D. M. Tripathi R. B. (2008). The life, death, and replacement of oligodendrocytes in the adult CNS. *J. Neurochem.* 107 1–19. 10.1111/j.1471-4159.2008.05570.x 18643793

[B77] Mecha M. Feliu A. Carrillo-Salinas F. J. Rueda-Zubiaurre A. Ortega-Gutiérrez S. de Sola R. G. (2015). Endocannabinoids drive the acquisition of an alternative phenotype in microglia. *Brain Behav. Immun.* 49 233–245. 10.1016/j.bbi.2015.06.002 26086345

[B78] Mecha M. Torrao A. S. Mestre L. Carrillo-Salinas F. J. Mechoulam R. Guaza C. (2012). Cannabidiol protects oligodendrocyte progenitor cells from inflammation-induced apoptosis by attenuating endoplasmic reticulum stress. *Cell Death Dis.* 3:e331. 10.1038/cddis.2012.71 22739983 PMC3388241

[B79] Menn B. Garcia-Verdugo J. M. Yaschine C. Gonzalez-Perez O. Rowitch D. Alvarez-Buylla A. (2006). Origin of oligodendrocytes in the subventricular zone of the adult brain. *J. Neurosci.* 26 7907–7918. 10.1523/JNEUROSCI.1299-06.2006 16870736 PMC6674207

[B80] Merkle F. T. Tramontin A. D. García-Verdugo J. M. Alvarez-Buylla A. (2004). Radial glia give rise to adult neural stem cells in the subventricular zone. *Proc. Natl. Acad. Sci. U.S.A.* 101 17528–17532. 10.1073/pnas.0407893101 15574494 PMC536036

[B81] Michalski J. P. Kothary R. (2015). Oligodendrocytes in a nutshell. *Front. Cell Neurosci.* 9:340. 10.3389/fncel.2015.00340 26388730 PMC4556025

[B82] Mifsud G. Zammit C. Muscat R. Di Giovanni G. Valentino M. (2014). Oligodendrocyte pathophysiology and treatment strategies in cerebral ischemia. *CNS Neurosci. Ther.* 20 603–612. 10.1111/cns.12263 24703424 PMC6493108

[B83] Miron V. E. Boyd A. Zhao J.-W. Yuen T. J. Ruckh J. M. Jennifer L. (2013). M2 microglia and macrophages drive oligodendrocyte differentiation during CNS remyelination. *Nat. Neurosci.* 16 1211–1218. 10.1038/nn.3469 23872599 PMC3977045

[B84] Miron V. E. Kuhlmann T. Antel J. P. (2011). Cells of the oligodendroglial lineage, myelination, and remyelination. *Biochim. Biophys. Acta* 1812 184–193. 10.1016/j.bbadis.2010.09.010 20887785

[B85] Mitew S. Hay C. M. Peckham H. Xiao J. Koenning M. Emery B. (2014). Mechanisms regulating the development of oligodendrocytes and central nervous system myelin. *Neuroscience* 276 29–47. 10.1016/j.neuroscience.2013.11.029 24275321

[B86] Molina-Holgado E. Vela J. M. Arévalo-Martín A. Almazán G. Molina-Holgado F. Borrell J. (2002). Cannabinoids promote oligodendrocyte progenitor survival: involvement of cannabinoid receptors and phosphatidylinositol-3 kinase/Akt signaling. *J. Neurosci.* 22 9742–9753. 10.1523/JNEUROSCI.22-22-09742.2002 12427829 PMC6757831

[B87] Moreno-Martet M. Feliu A. Espejo-Porras F. Mecha M. Carrillo-Salinas F. J. Fernandez-Ruiz J. (2015). The disease-modifying effects of a Sativex-like combination of phytocannabinoids in mice with experimental autoimmune encephalomyelitis are preferentially due to Δ^9^-tetrahydrocannabinol acting through CB_1_ receptors. *Mult. Scler. Relat. Disord.* 4 505–511. 10.1016/j.msard.2015.08.001 26590655

[B88] Murataeva N. Straiker A. Mackie K. (2014). Parsing the players: 2-arachidonoylglycerol synthesis and degradation in the CNS. *Br. J. Pharmacol.* 171 1379–1391. 10.1111/bph.12411 24102242 PMC3954479

[B89] Nishiyama A. Boshans L. Goncalves C. M. Wegrzyn J. Patel K. D. (2016). Lineage, fate, and fate potential of NG2-glia. *Brain Res.* 1638 116–128. 10.1016/j.brainres.2015.08.013 26301825 PMC4761528

[B90] Olah A. Szekanecz Z. Biro T. (2017). Targeting cannabinoid signaling in the immune system: “high”-ly exciting questions, possibilities, and challenges. *Front. Immunol.* 8:1487. 10.3389/fimmu.2017.01487 29176975 PMC5686045

[B91] Panikashvili D. Simeonidou C. Ben-Shabat S. Hanuš L. Breuer A. Mechoulam R. (2001). An endogenous cannabinoid (2-AG) is neuroprotective after brain injury. *Nature* 413 527–531. 10.1038/35097089 11586361

[B92] Pertwee R. G. (2007). Cannabinoids and multiple sclerosis. *Mol. Neurobiol.* 36 45–59. 10.1007/s12035-007-0005-2 17952649

[B93] Pertwee R. G. Howlett A. C. Abood M. E. Alexander S. P. Di Marzo V. Elphick M. R. (2010). International union of basic and clinical pharmacology. LXXIX. Cannabinoid receptors and their ligands: beyond CB(1) and CB(2). *Pharmacol. Rev.* 62 588–631. 10.1124/pr.110.003004 21079038 PMC2993256

[B94] Popescu B. F. Lucchinetti C. F. (2012). Pathology of demyelinating diseases. *Annu. Rev. Pathol.* 7 185–217. 10.1146/annurev-pathol-011811-132443 22313379

[B95] Priestley R. Glass M. Kendall D. (2017). Functional selectivity at cannabinoid receptors. *Adv. Pharmacol.* 80 207–221. 10.1016/bs.apha.2017.03.005 28826535

[B96] Pryce G. Riddall D. R. Selwood D. L. Giovannoni G. Baker D. (2015). Neuroprotection in experimental autoimmune encephalomyelitis and progressive multiple sclerosis by cannabis-based cannabinoids. *J. Neuroimmune. Pharmacol.* 10 281–292. 10.1007/s11481-014-9575-8 25537576

[B97] Ribeiro R. Yu F. Wen J. Vana A. Zhang Y. (2013). Therapeutic potential of a novel cannabinoid agent CB52 in the mouse model of experimental autoimmune encephalomyelitis. *Neuroscience* 254 427–442. 10.1016/j.neuroscience.2013.09.005 24036373

[B98] Richardson W. D. Kessaris N. Pringle N. (2006). Oligodendrocyte wars. *Nat. Rev. Neurosci.* 7 11–18. 10.1038/nrn1826 16371946 PMC6328010

[B99] Rom S. Persidsky Y. (2013). Cannabinoid receptor 2: potential role in immunomodulation and neuroinflammation. *J. Neuroimmune. Pharmacol.* 8 608–620. 10.1007/s11481-013-9445-9 23471521 PMC3663904

[B100] Ross R. A. (2003). Anandamide and vanilloid TRPV1 receptors. *Br. J. Pharmacol.* 140 790–801. 10.1038/sj.bjp.0705467 14517174 PMC1574087

[B101] Roth A. D. Nunez M. T. (2016). Oligodendrocytes: functioning in a delicate balance between high metabolic requirements and oxidative damage. *Adv. Exp. Med. Biol.* 94 167–181. 10.1007/978-3-319-40764-7_8 27714689

[B102] Ruparel N. B. Patwardhan A. M. Akopian A. N. Hargreaves K. M. (2011). Desensitization of transient receptor potential ankyrin 1 (TRPA1) by the TRP vanilloid 1-selective cannabinoid arachidonoyl-2 chloroethanolamine. *Mol. Pharmacol.* 80 117–123. 10.1124/mol.110.068940 21441412 PMC3127531

[B103] Saab A. S. Tzvetanova I. D. Nave K. A. (2013). The role of myelin and oligodendrocytes in axonal energy metabolism. *Curr. Opin. Neurobiol.* 23 1065–1072. 10.1016/j.conb.2013.09.008 24094633

[B104] Sampaio-Baptista C. Johansen-Berg H. (2017). White matter plasticity in the adult brain. *Neuron* 96 1239–1251. 10.1016/j.neuron.2017.11.026 29268094 PMC5766826

[B105] Sánchez A. J. García-Merino A. (2012). Neuroprotective agents: cannabinoids. *Clin. Immunol.* 142 57–67. 10.1016/j.clim.2011.02.010 21420365

[B106] Schiavon A. P. Bonato J. M. Milani H. Guimaraes F. S. Weffort de Oliveira R. M. (2016). Influence of single and repeated cannabidiol administration on emotional behavior and markers of cell proliferation and neurogenesis in non-stressed mice. *Prog. Neuropsychopharmacol. Biol. Psychiatry* 64 27–34. 10.1016/j.pnpbp.2015.06.017 26187374

[B107] Shao B. Z. Wei W. Ke P. Xu Z. Q. Zhou J. X. Liu C. (2014). Activating cannabinoid receptor 2 alleviates pathogenesis of experimental autoimmune encephalomyelitis via activation of autophagy and inhibiting NLRP3 inflammasome. *CNS Neurosci. Ther.* 20 1021–1028. 10.1111/cns.12349 25417929 PMC6492996

[B108] Shen M. Thayer S. A. (1998). Cannabinoid receptor agonists protect cultured rat hippocampal neurons from excitotoxicity. *Mol. Pharmacol.* 54 459–462. 10.1124/mol.54.3.459 9730904

[B109] Shouman B. Fontaine R. H. Baud O. Schwendimann L. Keller M. Spedding M. (2006). Endocannabinoids potently protect the newborn brain against AMPA-kainate receptor-mediated excitotoxic damage. *Br. J. Pharmacol.* 148 442–451. 10.1038/sj.bjp.0706755 16682966 PMC1751782

[B110] Simons M. Nave K. A. (2015). Oligodendrocytes: myelination and axonal support. *Cold Spring Harb. Perspect. Biol.* 8:a020479. 10.1101/cshperspect.a020479 26101081 PMC4691794

[B111] Solbrig M. V. Fan Y. Hermanowicz N. Morgese M. G. Giuffrida A. (2010). A synthetic cannabinoid agonist promotes oligodendrogliogenesis during viral encephalitis in rats. *Exp. Neurol.* 226 231–241. 10.1016/j.expneurol.2010.09.003 20832403 PMC2981070

[B112] Spatola M. Dalmau J. (2017). Seizures and risk of epilepsy in autoimmune and other inflammatory encephalitis. *Curr. Opin. Neurol* 30 345–353. 10.1097/WCO.0000000000000449 28234800 PMC5831325

[B113] Spitzer S. Volbracht K. Lundgaard I. Karadottir R. T. (2016). Glutamate signalling: a multifaceted modulator of oligodendrocyte lineage cells in health and disease. *Neuropharmacology* 110 574–585. 10.1016/j.neuropharm.2016.06.014 27346208

[B114] Sponsler J. L. Kendrick-Adey A. C. (2011). Seizures as a manifestation of multiple sclerosis. *Epileptic Disord.* 13 401–410. 10.1684/epd.2011.0468 22258045

[B115] Stockings E. Zagic D. Campbell G. Weier M. Hall W. D. Nielsen S. (2018). Evidence for cannabis and cannabinoids for epilepsy: a systematic review of controlled and observational evidence. *J. Neurol. Neurosurg. Psychiatry* 89 741-753. 10.1136/jnnp-2017-317168 29511052

[B116] Sun J. Fang Y. Chen T. Guo J. Yan J. Song S. (2013a). WIN55, 212-2 promotes differentiation of oligodendrocyte precursor cells and improve remyelination through regulation of the phosphorylation level of the ERK 1/2 via cannabinoid receptor 1 after stroke-induced demyelination. *Brain Res.* 1491 225–235. 10.1016/j.brainres.2012.11.006 23148948

[B117] Sun J. Fang Y. Q. Ren H. Chen T. Guo J. J. Yan J. (2013b). WIN55,212-2 protects oligodendrocyte precursor cells in stroke penumbra following permanent focal cerebral ischemia in rats. *Acta Pharmacol. Sin.* 34 119–128. 10.1038/aps.2012.141 23202804 PMC4086494

[B118] Takase H. Washida K. Hayakawa K. Arai K. Wang X. Lo E. H. (2018). Oligodendrogenesis after traumatic brain injury. *Behav. Brain Res.* 340 205–211. 10.1016/j.bbr.2016.10.042 27829126

[B119] Tauheed A. M. Ayo J. O. Kawu M. U. (2016). Regulation of oligodendrocyte differentiation: Insights and approaches for the management of neurodegenerative disease. *Pathophysiology* 23 203–210. 10.1016/j.pathophys.2016.05.007 27342760

[B120] Tomas-Roig J. Wirths O. Salinas-Riester G. Havemann-Reinecke U. (2016). The cannabinoid CB1/CB2 agonist WIN55212.2 promotes oligodendrocyte differentiation *in vitro* and neuroprotection during the cuprizone-induced Central Nervous System demyelination. *CNS Neurosci. Ther.* 22 387–395. 10.1111/cns.12506 26842941 PMC5067581

[B121] Trotter J. Karram K. Nishiyama A. (2010). NG2 cells: properties, progeny and origin. *Brain Res. Rev.* 63 72–82. 10.1016/j.brainresrev.2009.12.006 20043946 PMC2862831

[B122] Turner R. Vink R. (2007). Inhibition of neurogenic inflammation as a novel treatment for ischemic stroke. *Drug News Perspect.* 20 221–226. 10.1358/dnp.2007.20.4.1103527 17637934

[B123] van der Stelt M. Di Marzo V. (2005). Cannabinoid receptors and their role in neuroprotection. *Neuromolecular Med.* 7 37–50. 10.1385/NMM:7:1-2:037 16052037

[B124] Vemuri G. S. McMorris F. A. (1996). Oligodendrocytes and their precursors require phosphatidylinositol 3-kinase signaling for survival. *Development* 122 2529–2537. 8756297 10.1242/dev.122.8.2529

[B125] Vigano F. Dimou L. (2016). The heterogeneous nature of NG2-glia. *Brain Res.* 1638 129–137. 10.1016/j.brainres.2015.09.012 26388262

[B126] Wade D. T. Collin C. Stott C. Duncombe P. (2010). Meta-analysis of the efficacy and safety of Sativex (nabiximols), on spasticity in people with multiple sclerosis. *Mult. Scler.* 16 707–714. 10.1177/1352458510367462 20558502

[B127] Wen J. Ribeiro R. Tanaka M. Zhang Y. (2015). Activation of CB2 receptor is required for the therapeutic effect of ABHD6 inhibition in experimental autoimmune encephalomyelitis. *Neuropharmacology* 99 196–209. 10.1016/j.neuropharm.2015.07.010 26189763

[B128] Zecca L. Youdim M. B. Riederer P. Connor J. R. Crichton R. R. (2004). Iron, brain ageing and neurodegenerative disorders. *Nat. Rev. Neurosci.* 5 863–873. 10.1038/nrn1537 15496864

[B129] Zhang R. Chopp M. Zhang Z. G. (2013). Oligodendrogenesis after cerebral ischemia. *Front. Cell Neurosci.* 7:201 10.3389/fncel.2013.00201 PMC3810592 24194700

[B130] Zhang R. Zhang Z. Chopp M. (2016). Function of neural stem cells in ischemic brain repair processes. *J. Cereb. Blood Flow Metab.* 36 2034–2043. 10.1177/0271678X16674487 27742890 PMC5363673

[B131] Zhang Y. Aizenman E. DeFranco D. B. Rosenberg P. A. (2007). Intracellular zinc release, 12-lipoxygenase activation and MAPK dependent neuronal and oligodendroglial death. *Mol. Med.* 13 350–355. 17622306 10.2119/2007-00042.Zhang PMC1952666

[B132] Zhang Y. Wang H. Li J. Dong L. Xu P. Chen W. (2006). Intracellular zinc release and ERK phosphorylation are required upstream of 12-lipoxygenase activation in peroxynitrite toxicity to mature rat oligodendrocytes. *J. Biol. Chem.* 281 9460–9470. 10.1074/jbc.M510650200 16431921

